# Brachialis Muscle Rupture in a Pediatric Patient Followed Up by Ultrasound Examinations: A Rare Case Report

**DOI:** 10.1155/2022/3391350

**Published:** 2022-06-23

**Authors:** Akihiro Yamaji, Masafumi Uesugi, Hiroshi Kamada, Harumitsu Ichimura, Masashi Yamazaki

**Affiliations:** ^1^Department of Orthopedic Surgery, Ibaraki Seinan Medical Center Hospital, Sakai, Sashima District, Ibaraki 306-0433, Japan; ^2^Department of Orthopedic Surgery, University of Tsukuba Hospital, Ibaraki, Tsukuba, Amakubo 305-8576, Japan

## Abstract

Isolated brachial muscle injuries are relatively rare injuries and reportedly occur during forced elbow extension. Though commonly conservative treatment approach is adopted, the treatment criteria remain unclear. Here, we report the case of a patient who experienced functional recovery after conservative treatment for an isolated brachial muscle injury. The patient was an 8-year-old boy whose chief complaint was left elbow pain. The injury occurred when the patient fell while playing on gymnastics bars and bruised the palmar side of his left elbow on the bar. Owing to the pain in the left elbow, the patient came to our institution. There were no clear signs of deformities or swelling in the left elbow and no obvious tenderness. X-ray and computed tomography (CT) imaging examinations revealed no signs of a fracture or dislocation, and the patient was diagnosed with left brachialis muscle rupture based on magnetic resonance imaging (MRI). Although the brachialis muscle was complete ruptured, a healing tendency was seen on body surface ultrasound examinations over time, and the patient was treated conservatively. After 3 weeks of cast immobilization, the patient underwent range of motion exercises. Two months after the injury, there were no issues with elbow joint function in daily life activities and no limitations in range of motion. Here, MRI was used to diagnose brachialis muscle rupture, and ultrasound examinations were utilized to make treatment decisions.

## 1. Introduction

A closed rupture of the brachialis muscle is a rare injury [1–4]. Although some studies have reported this condition in adults, only a few studies have reported it in children [5]. It is observed from the past literature that the conservative approach had been preferred over surgical intervention; yet, the criteria for selecting the treatment method have not been clarified. Here, we report the case of a closed rupture of the brachialis muscle in a pediatric patient who was followed up using ultrasound examination.

## 2. Case Presentation

The patient was an 8-year-old boy, who had previously done gymnastics, was 125 cm tall, weighed 24.3 kg, and did not have any remarkable medical history.

While playing on bars, the patient fell and hit his left elbow on the bar. In the medical interview, the patient remembered hitting his left elbow but not the exact location of the injury. The patient was then referred to our institution.

Preliminary examination revealed a swelling located slightly proximal to the left elbow, and the patient complained of moderate pain in the same location; however, there was no apparent external bleeding or bruising of the skin. Flexion of the left elbow was possible with assistance, and active movement of the fingers was unrestricted. Palpation of the radial artery also showed unremarkable results.

**Figure 1:**
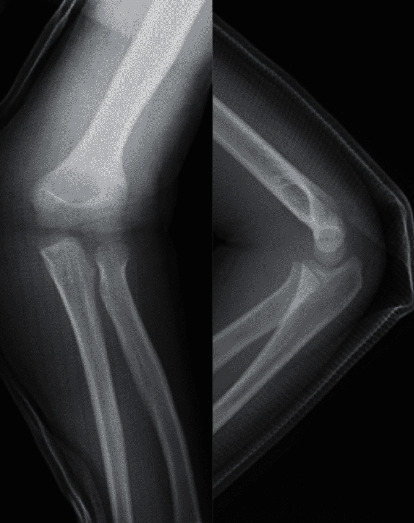
Plain X-ray images.

**Figure 2:**
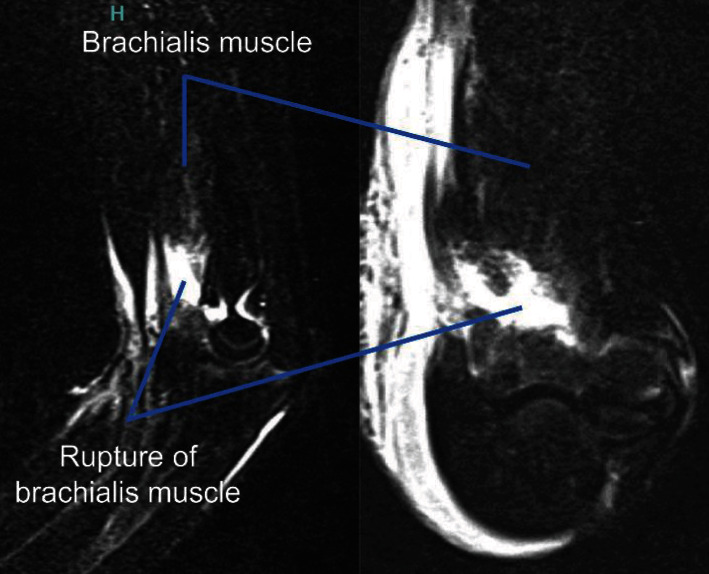
Short-TI inversion recovery magnetic resonance imaging. Brachialis muscle was ruptured completely.

**Figure 3:**
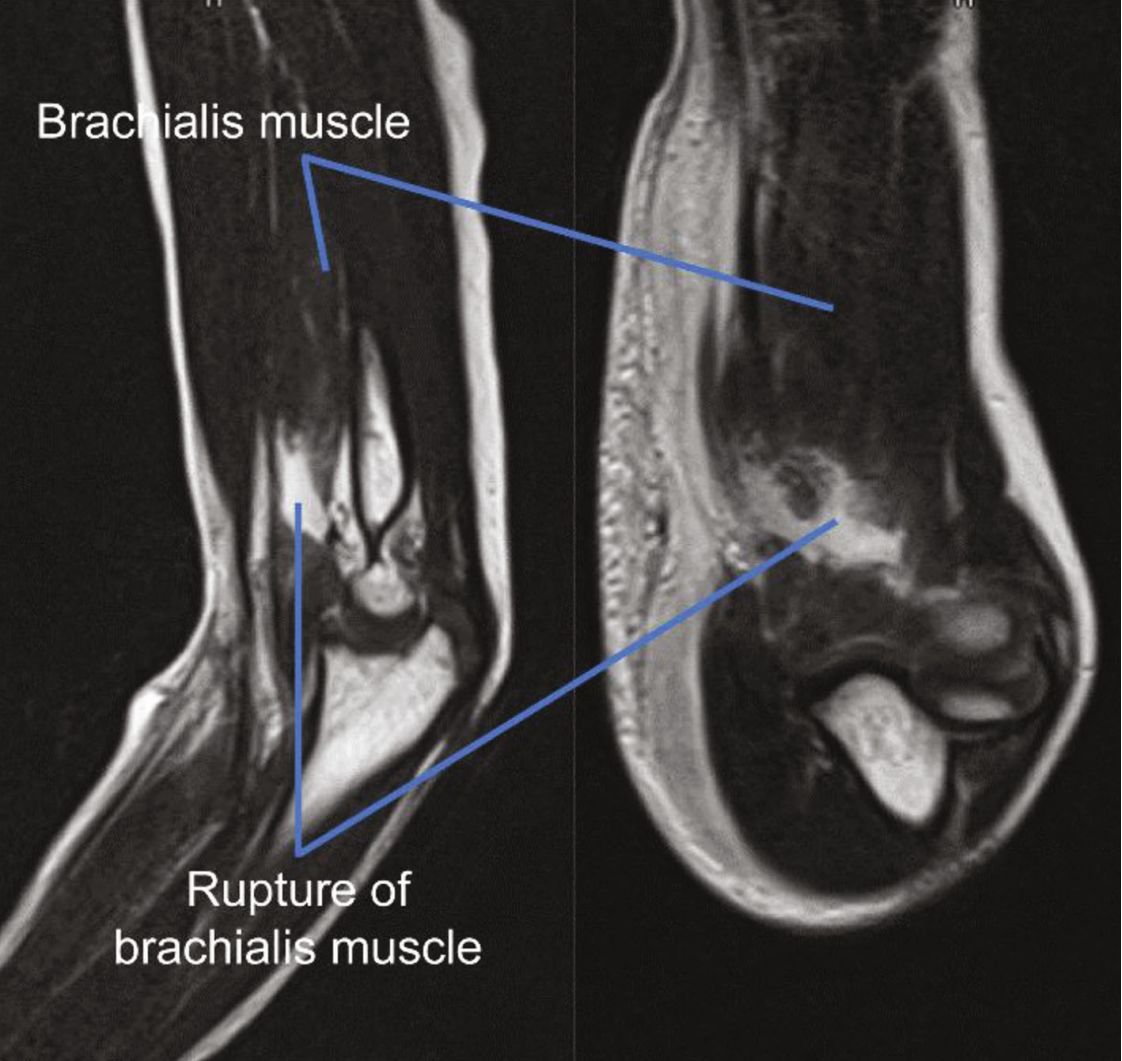
T2 magnetic resonance imaging.

We suspected that the patient had a supracondylar humerus fracture. However, plain X-ray imaging (Figure 1) and computed tomography (CT) did not show any fractures, dislocation, or epiphyseal injury. Hence, as we then suspected a soft tissue injury, we performed magnetic resonance imaging (MRI) (Figures 2 and 3) to check for a muscle rupture or an epiphyseal injury. MRI of the sagittal and axial slices showed an area of high signal intensity on short-TI inversion recovery in the deep layer of the tendon transfer of the biceps brachii and, as the continuity of the brachialis muscle was interrupted, the patient was diagnosed with brachialis muscle rupture.

Clinical decision-making was made to go with conservative methods rather than surgical options. On the same day, his left elbow was fixed with cast immobilization at 120° flexion of the elbow. We attempted to bring the brachial muscle rupture into contact by putting the elbow joint in the flexed position.

**Figure 4:**
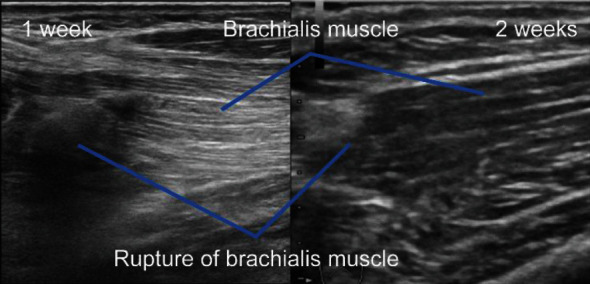
Ultrasound examinations (left: one week after the injury; right: two weeks after the injury).

One week after the injury, ultrasound (US) examinations with broadband (frequency band: 5–17 MHz and 7–12 MHz) small parts of linear array transducers (Figure 4, left) showed scar-like tissues in the area of the brachialis muscle rupture, which were considered as the signs of tissue repair. Subsequent ultrasound examinations were performed over time, and maintenance of continuity was confirmed (Figure 4, right).

### 2.1. Outcome

Ultrasound examinations at subsequent outpatient visits to examine the area of the brachialis muscle rupture confirmed that there were no findings of dehiscence in the area of the rupture. Cast immobilization was continued for 3 weeks. Following removal of cast, range of motion exercises of elbow was started. Six weeks after the injury, elbow range of motion was 30°–120°, and ultrasound examination showed continuity in the brachialis muscle, indicating recovery.

Three months after the injury, elbow range of motion improved to 30°–130°. The patient had no difficulties in performing activities of daily living using the involved elbow.

Eleven months after the injury, elbow range of motion improved further to 0°–135° and which is the same range as the healthy side. The patient was able to resume gymnastics 6 months after the injury. One year after the injury, we performed an MRI that showed complete continuity of the brachialis muscle.

## 3. Discussion

The brachialis muscle attaches proximally to the humeral shaft and distally to the ulnar tuberosity. Together with the biceps brachii and brachioradialis, these muscles control elbow joint flexion; furthermore, the brachial muscles is considered to be involved in elbow joint stabilization [6].

Thus far, only a few studies have reported brachialis muscle ruptures.

Notably, a study by Van den Berghe et al. in 2001 was the first to report the case of a 67-year-old man with a brachialis muscle rupture caused by lifting a statue of Christ [1].

To the best of our knowledge, there have been 10 case reports of this injury in physically active individuals such as athletes, with ages ranging from 8 to 67 years; additionally, the patients were predominantly male [1–10]. Reportedly, this injury was commonly caused by an external force forcing the extension of the elbow joint and when the elbow joint was bent due to lifting an object or was forced to extend when performing actions such as tackling in contact sports.

In 2020, Forsythe et al. reported a case of brachialis muscle rupture in an 8-year-old girl. The patient was reportedly injured while performing walking exercises with her back on the floor.

According to the extent of our search, this was the second case report of a brachialis muscle rupture in children [5]. We believe that the brachialis muscle ruptured due to a hyperextension injury in this patient. In terms of physical findings, owing to the fact that the brachialis muscle is located in the deep layer of the biceps brachii, palpation from the surface of the body is difficult, and MRI is considered a useful diagnostic modality [3].

Conservative and surgical treatments are reported as treatment options; however, there is no standard treatment policy. Among the 10 case reports, 9 were treated conservatively [1–10]. Surgical treatment was considered in only one patient who was a rugby player. This patient was highly physically active; hence, the tear was repaired surgically. In this study, we also used body surface ultrasound examinations to evaluate the ruptured area [7]. The dynamic continuity of the rupture was maintained throughout the follow-up period, and the repair course was considered favorable. However, surgical treatment should be considered if the tear of the rupture does not reduce in size [4].

Moreover, a study has reported using ultrasound to diagnose brachial muscle rupture. Previously, brachialis injuries were treated among immature patients using the MRI or contrast MRI as prognostic tool [5]. Despite the fact that it may be objectively more difficult to assess the outcomes of body surface ultrasound examinations than that of MRIs and that MRIs may be necessary for definitive diagnoses, body surface ultrasound examinations are necessary to evaluate the size of the tear and dynamic continuity in the area of the rupture [7]. Ultrasounds are also considered a simple and economically useful investigational method that could be used for evaluations over time.

The previous reports on the injury have not typically included details of immobilization in conservative treatment and subsequent range of motion exercises, and there have been some reports where cast immobilization was not performed. It was our understanding that cast immobilization of elbow joint would be needed to repair the tear, and we performed immobilization with a plaster slab for 3 weeks. Two months after the injury, the patient did not experience issues with elbow joint function in daily life activities; six months after the injury, the patient was able to resume gymnastics, showing favorable recovery.

Here, we report a case of closed brachialis muscle rupture caused by direct external force. The function of the elbow joint recovered favorably after 3 weeks of cast immobilization. Body surface ultrasound examination to assess the brachialis muscle rupture was a simple and useful method to evaluate the treatment course.

## Data Availability

The datasets generated during and/or analyzed during the current study are available from the corresponding author on reasonable request.
